# Supplementary material for: The Histone Methyltransferase SETD8 Regulates the Expression of Tumor Suppressor Genes via H4K20 Methylation and the p53 Signaling Pathway in Endometrial Cancer Cells
Source: Cancers (Basel). 2022 Oct 31;14(21):5367. doi: 10.3390/cancers14215367 (PMC9655767; doi:10.3390/cancers14215367)
Supplement: Supplementary file 1 [file cancers-14-05367-s001.zip › cancers-1962511-Supplementary Figures.pdf]

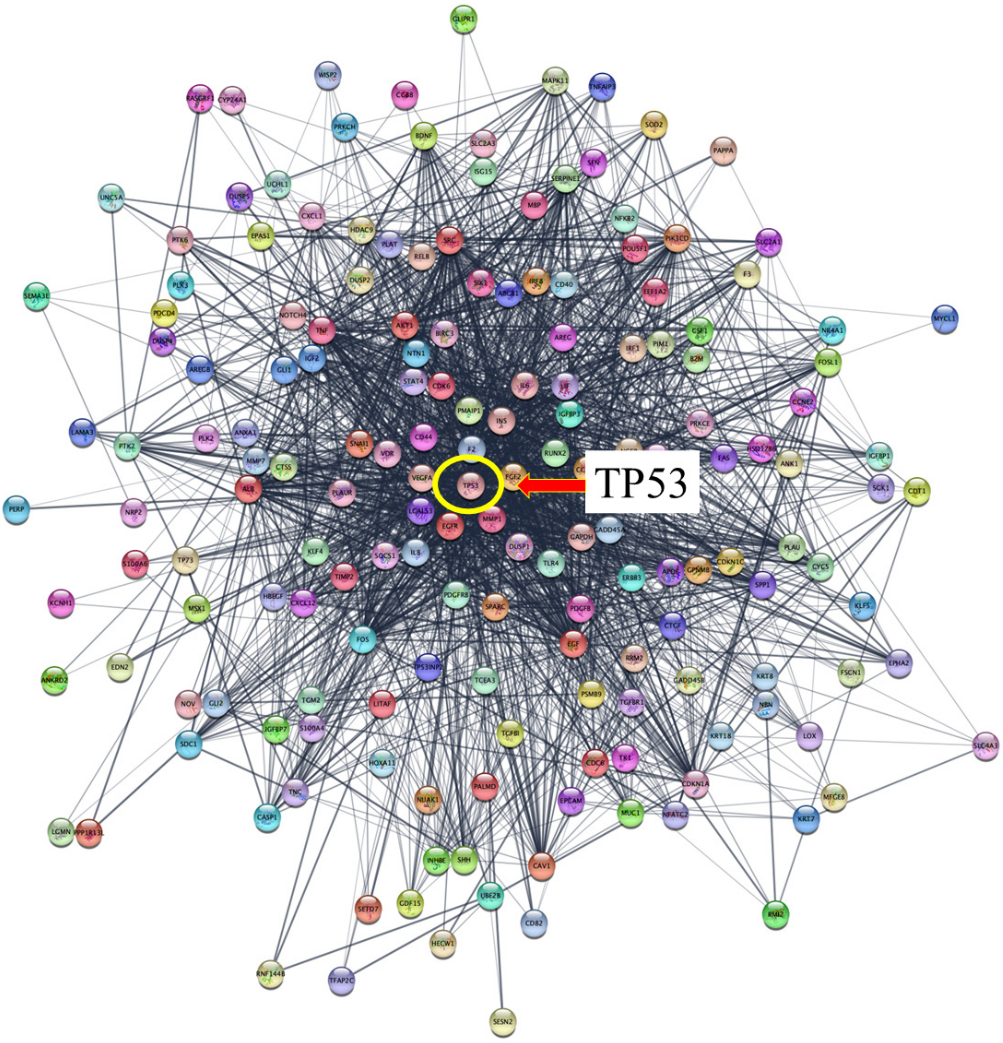

Figure S1. Protein-protein interactions (PPIs) of DEGs in SETD8 knockdown HEC50B cells. PPIs were visualized by Cytoscape with string database. The data is the same as Figure 3D, but an expanded version is included for ease of understanding.

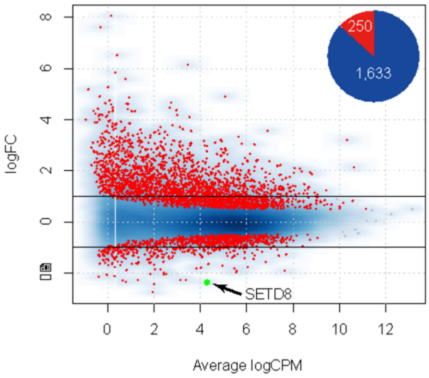

Figure S2. MA plots showing differentially expressed genes (DEGs: false discovery rate (FDR) < 0.05, red dots) between HEC1B cells treated with negative control (n = 3) and SETD8 targeting siRNA (n = 4). The differentially expressed SETD8 gene is indicated by the arrow (green dot). The black lines indicate the log2 fold change (logFC) at 1 and −1. Average logCPM: the average log2 count per million. The circle plot shows the number of DEGs.
