# Supplementary figures and images for: The Histone Methyltransferase SETD8 Regulates the Expression of Tumor Suppressor Genes via H4K20 Methylation and the p53 Signaling Pathway in Endometrial Cancer Cells
Source: Cancers (Basel). 2022 Oct 31;14(21):5367. doi: 10.3390/cancers14215367 (PMC9655767; doi:10.3390/cancers14215367)

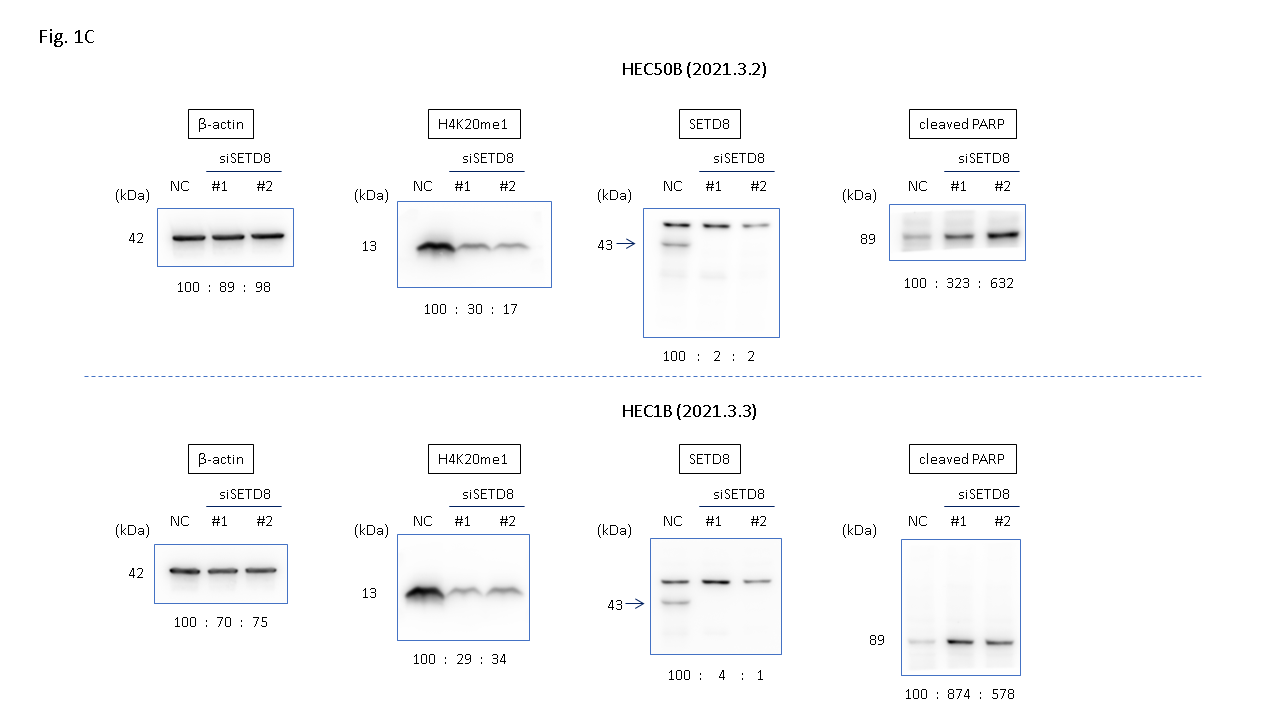

Supplement: Supplementary file 1 [file cancers-14-05367-s001.zip › uncropped WB data of Figure 1C.TIF]

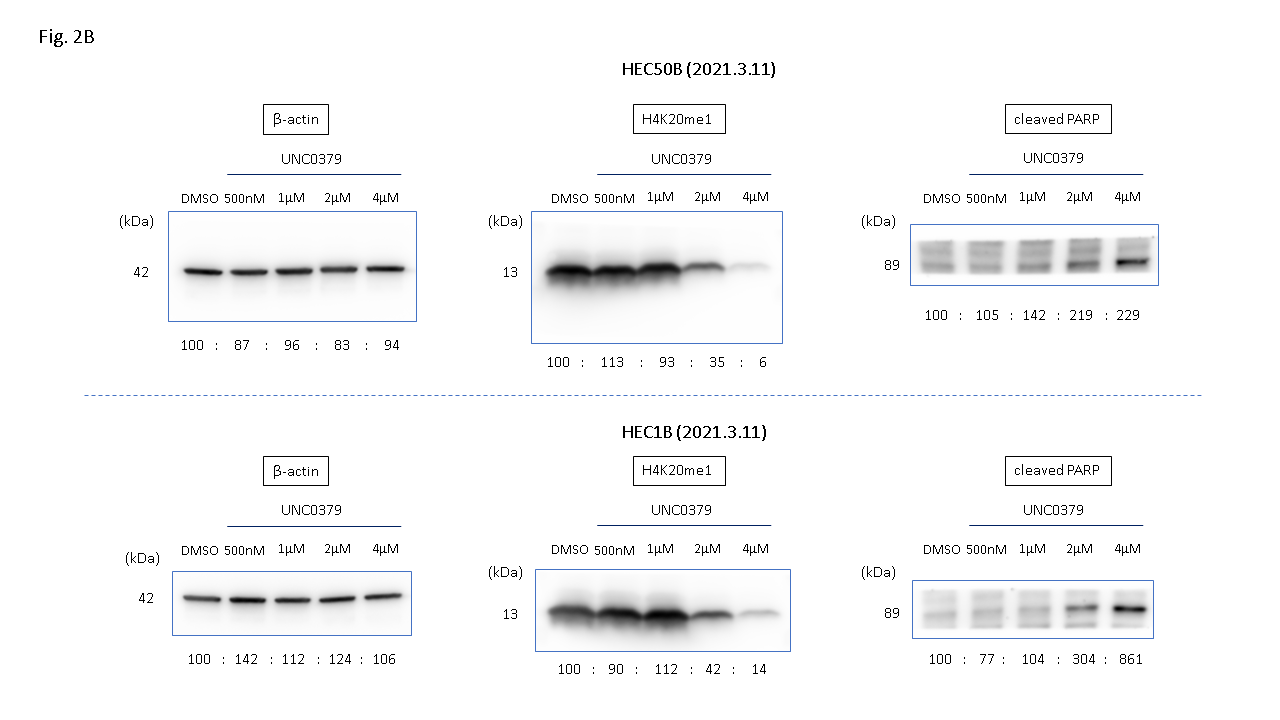

Supplement: Supplementary file 1 [file cancers-14-05367-s001.zip › uncropped WB data of Figure 2B.TIF]

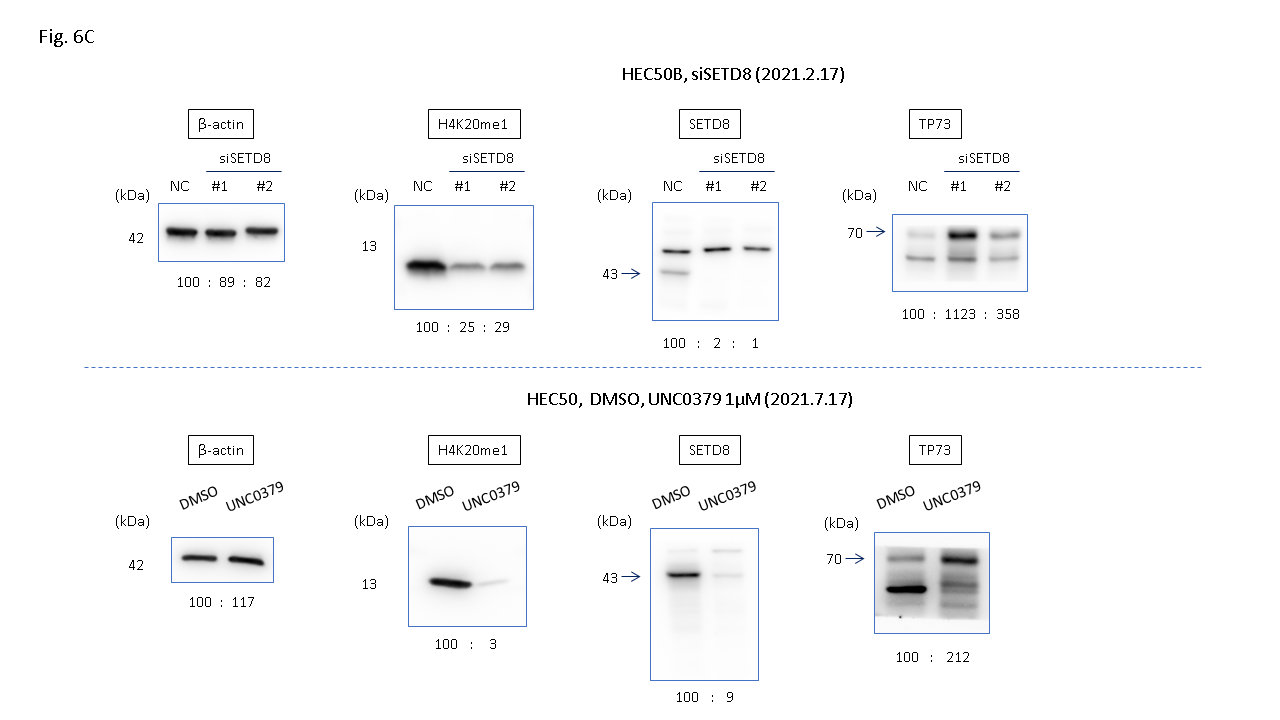

Supplement: Supplementary file 1 [file cancers-14-05367-s001.zip › uncropped WB data of Figure 6C.TIF]
